# A New Hybrid Approach Based on Time Frequency Images and Deep Learning Methods for Diagnosis of Migraine Disease and Investigation of Stimulus Effect

**DOI:** 10.3390/diagnostics13111887

**Published:** 2023-05-28

**Authors:** Fırat Orhanbulucu, Fatma Latifoğlu, Recep Baydemir

**Affiliations:** 1 Department of Biomedical Engineering, Inonu University, Battalgazi 44000, Turkey; 2 Department of Biomedical Engineering, Erciyes University, Kayseri 38039, Turkey; 3 Department of Neurology, Erciyes University, Kayseri 38039, Turkey; rbaydemir@erciyes.edu.tr

**Keywords:** convolutional neural networks, electroencephalogram, migraine disease, continuous wavelet transform, short-time Fourier transform

## Abstract

Migraine is a neurological disorder that is associated with severe headaches and seriously affects the lives of patients. Diagnosing Migraine Disease (MD) can be laborious and time-consuming for specialists. For this reason, systems that can assist specialists in the early diagnosis of MD are important. Although migraine is one of the most common neurological diseases, there are very few studies on the diagnosis of MD, especially electroencephalogram (EEG)-and deep learning (DL)-based studies. For this reason, in this study, a new system has been proposed for the early diagnosis of EEG- and DL-based MD. In the proposed study, EEG signals obtained from the resting state (R), visual stimulus (V), and auditory stimulus (A) from 18 migraine patients and 21 healthy control (HC) groups were used. By applying continuous wavelet transform (CWT) and short-time Fourier transform (STFT) methods to these EEG signals, scalogram-spectrogram images were obtained in the time-frequency (T-F) plane. Then, these images were applied as inputs in three different convolutional neural networks (CNN) architectures (AlexNet, ResNet50, SqueezeNet) that proposed deep convolutional neural network (DCNN) models and classification was performed. The results of the classification process were evaluated, taking into account accuracy (acc.), sensitivity (sens.), specificity (spec.), and performance criteria, and the performances of the preferred methods and models in this study were compared. In this way, the situation, method, and model that showed the most successful performance for the early diagnosis of MD were determined. Although the classification results are close to each other, the resting state, CWT method, and AlexNet classifier showed the most successful performance (Acc: 99.74%, Sens: 99.9%, Spec: 99.52%). We think that the results obtained in this study are promising for the early diagnosis of MD and can be of help to experts.

## 1. Introduction

Migraine is a neurological disorder that occurs as a result of symptoms originating from the vessels and nerves in the brain [1]. MD is one of the most common neurological diseases [2]. It ranks sixth among the most common diseases in the world [3]. The most common symptoms of MD are severe headaches, nausea, vomiting, and sensitivity to sound and light [4]. A migraine patient may have attacks once or twice a month. For this reason, it is important to be able to diagnose MD. MD can be diagnosed and analyzed by experts based on clinical data. However, the manual interpretation of EEG signals by experts can be cumbersome and time-consuming [5]. For this reason, Computer Aided Diagnosis (CAD) systems that can support experts in this regard are important. CAD systems are computer-based systems that can help experts by making quick decisions. Thanks to CAD systems, the workload can be lightened and experts can be supported in the final diagnosis. Studies on the development of these systems should continue.

MD can be estimated based on clinical data. In addition to these data, several neuroimaging (magnetic resonance imaging, etc.) methods are also used to support the diagnosis of MD. However, EEG is the most effective method and is preferred in clinical fields due to its low hardware requirement and low cost compared to neuroimaging techniques. EEG, which can yield important information for the diagnosis of neurological diseases and is especially very popular in the field of neuroscience, can provide convenience to specialist physicians in the diagnosis of diseases [5,6]. Neuro-spike signals provide a more detailed perspective on individual neuron activity, allowing for the analysis of axonal transmission and temporal modulation in neural communication. However, EEG signals offer a broad overview of brain activity, making them suitable for detecting neurological disorders and studying overall brain dynamics. EEG signals are stationary and non-linear. For this reason, analysis of EEG signals can be difficult. By applying signal processing methods to EEG signals, which are difficult to analyze, studies are carried out on the diagnosis of neurological diseases, including some feature extraction processes [5,7]. In addition to signal processing methods, it is possible to easily analyze EEG signals by applying machine learning (ML) or DL methods [8]. According to recent studies, when applying these methods, CAD systems are recommended. In recent years, studies on neurological disorders have applied ML and DL methods together with signal processing methods [7,8,9,10].

DL is a new branch of machine learning that has received widespread attention in relation to the task of classifying EEG signals [11]. DL models can be comparatively more advantageous than ML methods because they can learn features automatically. In addition, when the appropriate number of data is used in studies with EEG signals, DL models can perform better than ML [12]. For this reason, EEG-based DL studies are promising and increasing in number [13]. EEG signal- and DL model-based studies are of interest in the diagnosis of neurological diseases. EEG signal-, ML-, and DL-based methods have been used with respect to the diagnosis of MD. However, studies regarding migraines and the use of EEG are generally scarce [11]. In particular, studies on the automatic diagnosis of MD using DL are negligible [14]. As a result of the application of ML and DL models to EEG signals, important studies in the literature on MD diagnosis have been reviewed and are summarized below.

- Akben et al. [6] analyzed the EEG signals obtained from migraine patients and the HC group under flash stimulation in their study and detected MD with an accuracy of 85% using the Support Vector Machine (SVM) classifier.
- Aslan separated the signals into subbands by applying the Tunable Q-Factor Wavelet Transform (TQWT) method to the EEG signals in his studies on the diagnosis of MD. By extracting features from these subbands, the classification process between MD and HC was performed using the Rotation Forest algorithm. As a result of the classification process, an accuracy rate of 89.6% was obtained [10].
- In another study, Aslan applied the Empirical Mode Decomposition (EMD) method to EEG signals and separated the signals into subbands. As a result of the classification process using the features extracted from these subbands, an accuracy rate of 92.7% was obtained using a Random Forest (RF) algorithm [15].
- In another similar study, Subaşı et al., using Discrete Wavelet Transform (DWT) and RF algorithm, distinguished MD from the HC group with an accuracy of 85.95% [16].
- In a study conducted for clinical support purposes, Yin et al. [17] succeeded in distinguishing between tension-type headaches and migraine with 90% accuracy as a result of the system they developed based on the K-Nearest Neighbors (KNN) algorithm.
- In terms of the studies that used DL, Göker [14] created feature vectors by applying the Welch method to EEG signals. She used several ML methods and a Bidirectional Long-Short Term Memory (BiLSTM) model in the classification phase. The most successful performance was achieved using the BiLSTM model, as it succeeded in classifying the MD and HC groups with 95.99% accuracy.

In this study, a new EEG-based hybrid system is proposed for the automatic diagnosis of MD using signal processing methods and DL models. This proposed system aimed to determine the system that shows the most successful performance in diagnosing MD by applying different signal processing methods and classifiers. Migraine is one of the most common neurological diseases [2,3]. However, EEG-based studies on migraine are limited [11]. For this reason, our motivation for this study was that there are almost no studies on the diagnosis of MD using EEG signals, especially with DL models [11,14]. We think that DL studies on the diagnosis of migraine disease are scarce and that this study is important as it fills a gap in the literature. In addition, the fact that the data set used in the study is new (2020) and has been used very little until this research has encouraged us to conduct this study. The purpose, summary, and contributions of this study can be explained as follows:

1. In this study, a new system based on EEG and DL that can support specialists in the automatic and early diagnosis of MD is proposed.
2. For this purpose, in this study, recorded EEG signals based on the resting state (R), visual stimulus (V), and auditory stimulus (A) from MD and HC groups were analyzed. As a result of the analyses made possible by signal processing methods and DL models, MD and HC groups were able to be classified. This study aims to be original and to contribute to future studies.
3. In this study, 1-D EEG signals obtained from MD and HC groups were preprocessed and noise-free. The noise-free EEG signals were transformed into scalogram-spectrogram images in the time-frequency domain by using the ‘CWT and STFT’ T-F transform methods.
4. The classification was carried out by applying the scalogram and spectrogram images of the MD and HC groups to some CNN architectures (AlexNet, ResNet50, SqueezeNet) and to the DCNN model that we created ourselves.
5. The effect of the stimuli was also examined by performing the classification process in three situations (R-A-V). As a result of the classification process, both the performance comparison of CWT and STFT, which are signal processing methods, and the performance of the DL models used in the study were compared. In this way, the best-performing state, method, and classifier model were determined. Regarding the use of EEG signals, DL, and ML in the diagnosis of MD, as far as we know, this study is the first of its kind compared to similar studies in the literature.

In the Section 2 of the study, information about the dataset, preprocessing step, signal processing methods, and DL models are given. In the Section 3, the results of the study and the interpretation of the results are given. In the Section 4, the results obtained are compared with the results of similar studies, and the contributions of this study to the literature and the limitations of the study are discussed. In the Section 5, conclusions are drawn and considerations for future studies are given.

## 2. Methodology

**Figure 1:**
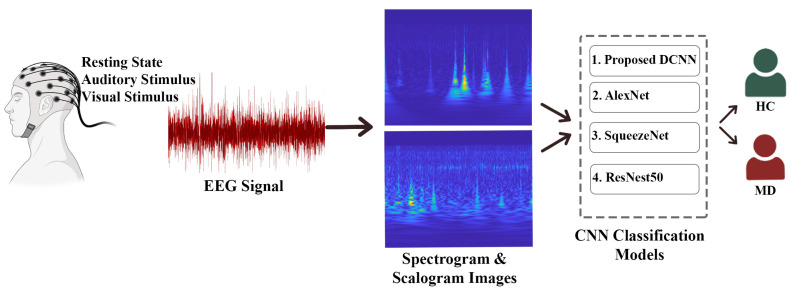
Flowchart of the proposed study for the diagnosis of migraine disease.

In this part of the study, information is given about the data set and the methods used in the proposed model. In this study, a new EEG-based hybrid system for the automatic diagnosis of MD is proposed as a result of applying signal processing methods and CNN models to EEG signals. The processes applied in the proposed system are summarized below, and a flow chart of the study is shown in Figure 1.

- In the preprocessing step, the noises in the recorded EEG signals for visual, auditory stimulus, and resting state obtained from the multi-channel were cleaned using a 0.5–40 Hz finite impulse response (FIR) filter.
- Scalogram and spectrogram images were created in the T-F plane of the signals by applying the ‘CWT and STFT’ T-F transform methods for the signal processing of noise-free EEG signals.
- In this study, scalogram and spectrogram images were applied for the first time to CNN architectures (AlexNet, ResNet50, SqueezeNet) and the proposed DCNN model for three states (R-A-V). The classification process analyzed the MD–HC groups for the three situations and the applied methods. Classification performance criteria (Acc., Sens., and Spec.) ratios were obtained and interpreted for all situations and methods applied in the study.

### 2.1. Participants and Dataset

The dataset of EEG signals used in this study is was created recently and publicly shared by Carnegie Mellon University [18]. EEG signals were obtained from 21 HC groups (12 females/9 males; all 19–54 years old; mean age 27.9 years) without a headache and 18 migraine patients in the interictal period (13 females/5 males; all 19–54 years old; mean age 27.6) was recorded. Subjects participating in the study were selected according to the criteria of the International Headache Society. EEG signals have a sampling frequency of 512 Hz and were recorded from 128 channels [18]. EEG recordings were obtained using the BioSemi Active Two system. EEG recordings were taken by sending audio-visual stimuli to the subjects according to their resting state. For the visual state, the grid pattern with the contrast adjustment changed at a frequency of 4 Hz or 6 Hz. For the auditory stimulus, auditory tones with a frequency of 4–6 Hz were recorded. In the resting state, they were asked to focus on the fixed plus sign on the screen [19]. In this study, all three situations were analyzed, and the results were compared. Detailed information about the dataset and experimental setup can be found in refs. [18,19].

### 2.2. Signal Preprocessing and Time-Frequency Transform Techniques

**Figure 2:**
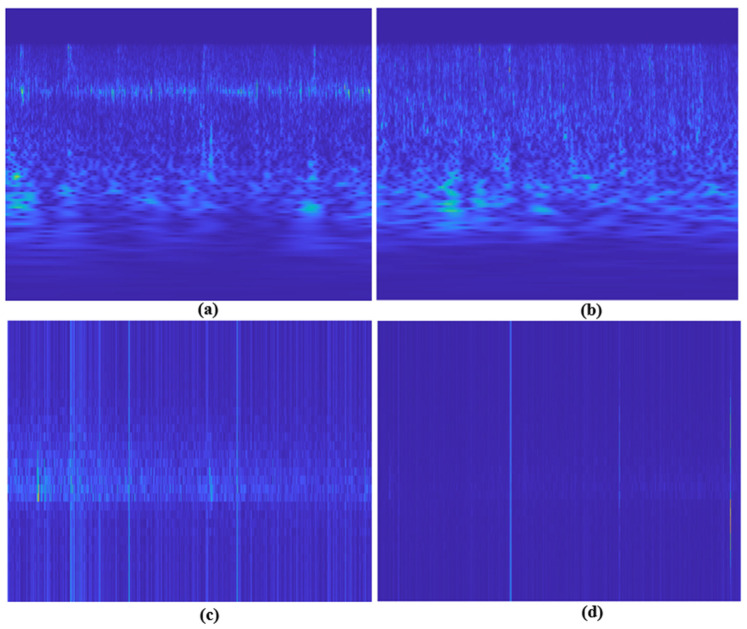
Sample CWT (**a**) and sample STFT (**c**) images of migraine patients. Sample CWT (**b**) and sample STFT (**d**) images of healthy control group.

In the first stage of this study, the FIR filter (0.5–40 Hz) was preferred in the preprocessing stage to clean the noises in the EEG signals used. FIR filters are easy to implement. It is also widely used due to its linear phase property and frequency stability [20]. In addition, 2 times downsampling was applied to the EEG signals, and the sampling frequency was set to 256 Hz. This helped reduce the processing load. After the preprocessing stage, CWT and STFT from T-F transform methods were applied to the noise-free signals to facilitate the analysis of the EEG signals and capture the details simultaneously. EEG signals contain oscillating and fluctuating frequency components. In order to obtain more information from oscillating and non-stationary signals such as EEG, T-F transform methods are applied to generate T-F representations of the signal. Thanks to these methods, the relationship between the time and frequency properties of the signal can be examined. It has been stated that images in the T-F plane obtained from non-stationary physiological signals such as EEG can be used with deep learning models [21]. For this reason, scalogram and spectrogram images were obtained in the T-F plane by using the MATLAB software program thanks to the transformation techniques applied in this study. Sample images obtained from the MD and HC groups are given in Figure 2. These images have been adjusted to the appropriate input sizes according to the models in the classification process and made ready for use as data in classification models.

#### 2.2.1. Continuous Wavelet Transform

The CWT method is a suitable and preferred method for the analysis of non-stationary signals that vary with time and scale [22]. The CWT method can provide appropriate time-frequency resolutions and capture transients in the EEG signal with a high temporal resolution [23]. Many wavelets can be used in the CWT method (Morlet–Morse–Bump wavelet). In this study, CWT wavelets were tested, and the Bump wavelet, which gave the most successful result for all three cases, was preferred. As a result of applying the CWT method to EEG signals, scalogram images were obtained. The formula of the CWT method is shown in Equation (1). In the equation, ‘*x*(*t*)’ represents the EEG signal in the time axis, *ψ*(*t*) represents the main wavelet, and *a* and *b* are the parameters [22,23].

(1) $$W_{a,\;b\;}\left[x\left(t\right)\right]=\frac{1}{\surd a}\int_{-\infty }^{\infty }x\left(t\right)\;\psi^{*}\left(\frac{t-b}{a}\right)dt.$$

#### 2.2.2. Short-Time Fourier Transform

The STFT method is an improved version of the Fourier method. In this method, the signals in the time domain are divided into blocks and the Fourier transform is evaluated in each block. The STFT method, also known as the windowed Fourier transform, also acts as a symmetric band-pass filter. STFT method is one of the most popular T-F analysis methods preferred in studies and compared with CWT [24]. As a result of applying the STFT method to EEG signals, spectrogram images were obtained. The STFT transformation is shown in Equation (2). In Equation (2), ‘*x*(*t*)’ represents the signal and ‘*w*(*t*)’ is the window function. The length of the windows for each block is equal, and the *x*(*t*) signal is assumed to be stationary within the window time [25]. The spectrogram of the *X*(*t*) signal can also be defined as $\left({\left|X_{\;}\left(t,\;f\right)\right|}^{2}\right)$.

(2) $$X_{\;}\left(t,\;f\right)=\int_{-\infty }^{\infty }x\left(\tau \right)w\left(\tau -t\right)exp^{-j2\pi f\tau }d\tau .$$

### 2.3. Deep Learning Models

DL is a ML method consisting of neural networks that enable data properties to be learned sequentially [26]. In DL methods, features are learned automatically. In contrast to the use of ML methods, there is no need to pre-extract features. For this reason, DL methods seem superior to ML methods [26]. DL models are of great interest in the classification of EEG signals and in the diagnosis of neurological diseases [11]. CNN is the most widely used of these models. CNN models are preferred in this study because DL models can perform feature selection automatically and generally perform better than ML methods. In the classification phase of this study, AlexNet, ResNet50, and SqueezeNet, which are commonly used CNN architectures, were preferred. In addition to these architectures, the DCNN model we recommend was used in the classification phase. In this way, the performances of the preferred CNN architectures and the proposed DCNN classifier model were compared. Information about the preferred architectures and the proposed DCNN model in the study are explained in Section 2.3.1.

#### 2.3.1. Convolutional Neural Networks and the Proposed DCNN Model

CNN-based models are seen as one of the most popular deep learning techniques. They consist of multiple layers and are used for feature extraction and classification [27]. In general, neural networks consist of an input layer, one or more hidden layers, and an output layer. CNN-based models are DL techniques consisting of network layers and have become popular in recent years for the classification of signals or images and object recognition [28,29,30]. In addition, CNN models are generally seen as the best DL networks and are frequently preferred in the classification of medical images and biomedical signal processing studies [31]. In the CNN method, several parameters may need to be regulated in the architectures we have designed, and this process can be time-consuming. For this reason, in some studies, well-designed CNN architectures such as AlexNet and DenseNet are preferred at the classification stage [32]. Information in a CNN-processed raw image is preserved. In an image applied as an input to the CNN model, the information between the pixels is included in the networks [30,32]. There are many parameters that need to be adjusted when designing the CNN model. CNN generally consists of three layers: the convolution, pooling, and fully connected layers.

i. The convolution layer is the basic building block of the convolutional network and contains filters that are set during the training process. It is the layer responsible for producing the output of each neuron in the input layer. The final output of the convolution layer is a vector [29,32,33].
ii. The pooling layer can protect the network by subsampling the output of the convolution layer. By reducing the amount of parameters and calculations in the network, mismatch in the network is controlled and overfitting can be avoided [28,32,33].
iii. The fully connected layer is where the classification process takes place. Neurons in this layer are associated with all activations in the previous layer [28,32,33].

**Figure 3:**
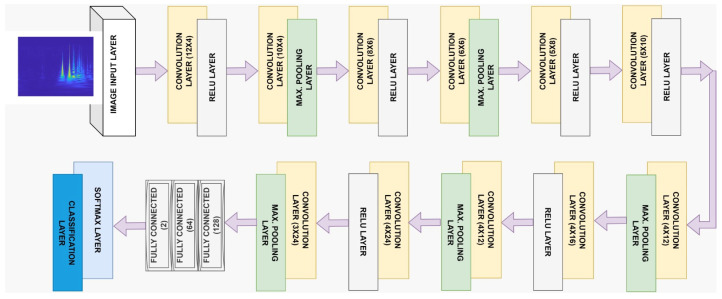
The architecture of the DCNN model proposed in the study for the diagnosis of migraine disease.

In this study, AlexNet, ResNet50, and SqueezeNet from CNN architectures were used. Detailed information about these architectures can be found in Ref. [33]. These architectures were created by using the layers accepted in the literature and preferred in studies. In addition to these architectures, a DCNN model, whose layers we created ourselves, is proposed. While creating the DCNN architecture, different layers and parameters were trialed many times. As a result of these trials, the layers and parameters of the DCNN model that gave the most successful result were determined. The DCNN model with the most successful performance was created. The initial learning rate of the model is 0.0001; the max epochs are 12. The mini-batch size was set to 64, and Adam was chosen as the optimizer. In this study, the proposed DCNN model for the detection of migraine disease consists of an Input layer, Convolution layer, ReLU layer, Max Pooling layer, Fully Connected layer, Softmax layer, and Classification layers. The layer information and architecture of the proposed model are given in Figure 3. In the data preparation part, before the classification process, the CWT and STFT equivalents of the EEG signals of 39 individuals (21 HC-18 MD) were obtained from 64 channels, and the data were made ready for the classifier input. The images obtained as a result of T-F transformation techniques and used as data in the classification stage were adjusted to appropriate input sizes according to the models. For the proposed DCNN model, the input image size is set to 256 × 256 × 3. Input sizes for AlexNet and SqueezeNet models are 227 × 227 × 3. For ResNet50, the input dimensions are set to 224 × 224 × 3. As a result of these processes, MD and HC groups were classified.

### 2.4. Classification Process and Performance Evaluation Metrics

**Figure 4:**
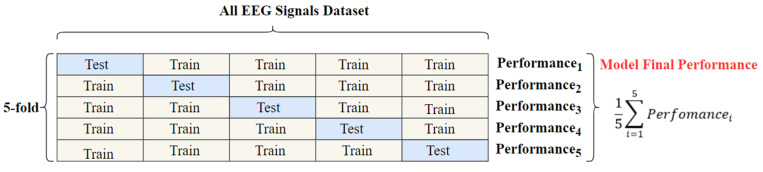
Sample diagram of the preferred five-fold cross-validation method for classification.

In this study, the models described in Section 2.3.1 were used to classify the MD and HC groups. In the classification process, images in the T-F plane obtained from the EEG signals as a result of the methods mentioned in Section 2.2 were used as data. Classification stages were carried out using the MATLAB software program. In the classification process, the k-fold cross-validation (CV) technique was applied. In the K-fold CV technique, the data is divided into k equal parts. K-1 of the parts is used to train the model, and the remaining part is used for the testing phase of the model. These stages continue by repeating k times, and the performance of the model is determined by obtaining the average of the results. Thus, possible deviations and errors are minimized. In this study, CV: is set as 5. According to the CV:5 process, 20% of the data was set to be tested and 80% to be trained, and the classification process was carried out. As a result of each fold operation, acc., sens., and spec., which comprise the performance criteria evaluated in the study, were calculated. The performances of the classifier models were calculated by taking the average of these values. The diagram of the CV:5 technique is shown in Figure 4.

**Figure 5:**
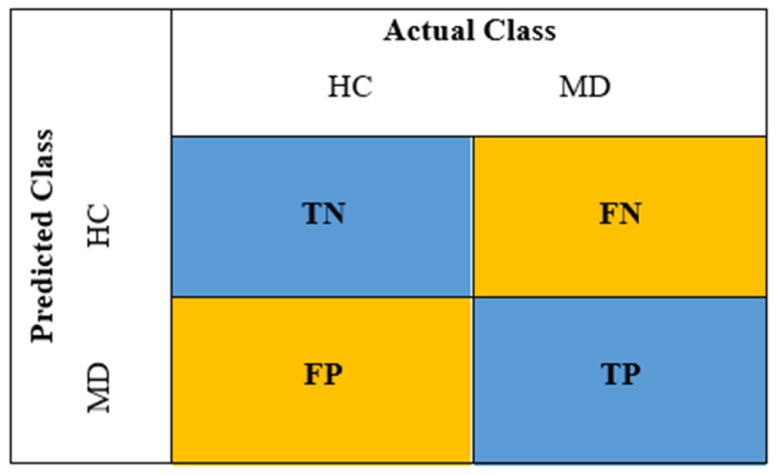
Sample confusion matrix used in the proposed study.

As a result of the classification process, the acc., sens. and spec. ratios, which comprise the performance criteria evaluated in the study, were calculated according to the sample Confusion Matrix given in Figure 5. During the calculation process, true positive (TP), true negative (TN), false positive (FP), and false negative (FN) rates were used. Acc., sens., and spec. calculations are given in Equations (3)–(5).

- The TP is the number of data predicted by the model in the MD class that is actually in the MD class.
- FP is data that does not actually belong to the MD class but that the model mistakenly predicts to belong to the MD class.
- TN is the number of data that is actually in the HC group, correctly predicted by the model as belonging to the HC group.
- FN is the number of data that actually belongs to the MD class but is incorrectly predicted by the model as belonging to the HC group.

(3) $$Accuracy=\frac{\left(TP+TN\right)}{\left(TP+TN+FP+FN\right)}\times 100.$$

(4) $$Sensitivity=\frac{TP}{\left(TP+FN\right)}\times 100.$$

(5) $$Specificity=\frac{TN}{\left(TN+FP\right)}\times 100.$$

## 3. Experimental Results

Manual analysis of non-stationary physiological signals such as EEG can be difficult. While analyzing these signals using traditional methods, steps such as feature extraction, feature selection, and classification are required [34]. These steps can be laborious and time-consuming. To alleviate this, DL models that can automatically extract features and perform classification are preferred. For this reason, several CNN architectures and the DCNN model we created were preferred in this study. In this study, a new hybrid system based on EEG signal and DL model, which can support experts by providing an automatic diagnosis of MD, is proposed.

In the proposed system, auditory-visual stimuli from 18 MD and 21 HC groups and EEG signals recorded according to their resting state were used. The noises of these signals were cleaned in the preprocessing stage. Scalogram and spectrogram images in the T-F plane were obtained by applying CWT and STFT and the T-F transform methods to noiseless 1-D EEG signals. We aimed to capture the transient moments of non-stationary EEG signals by providing high-resolution images. The images obtained as a result of T-F transformation techniques and used as data in the classification phase were adjusted to the appropriate input sizes according to the classifier models. Then, these data were applied as inputs into AlexNet, SqueezeNet, ResNet50, and suggested DCNN models from CNN architectures, and classification was performed. A total of 2496 scalogram-spectrogram images obtained from 64 channels from 39 participants were used in the classification process. MD and HC groups were classified by applying the procedures described in Section 2.4.

The classification was performed separately for the CWT, STFT methods, and three states (R-A-V). In this way, while the performances of the models used in the classification were compared, the performance of both the methods and the three states were also compared. As a result of the classification process, acc., sens., and spec. values were obtained and interpreted. The results obtained with the CWT and STFT methods and DL models for the resting state are given in Table 1. While the results of the auditory stimulus status are given in Table 2, the results of the visual stimulus status are given in Table 3.

This study should be considered both as a comparison of methods and as a comparison of the states of resting, auditory, and visual stimuli. For the comparison of the methods used in this study, the performance of CWT and STFT methods in DL models was examined. In the same way, the performance results in the DL models were obtained by examining the stimulus states separately. In this way, the most successful method, classifier, and state were determined.

If we consider the performances of the methods used in the study, when Table 1, Table 2 and Table 3 were examined, it was observed that the CWT method was more successful than the STFT metho—according to the classifier performance criteria. The CWT method performed slightly better than the STFT method in all classifiers preferred in the study. In the CWT method, the highest accuracy rate was obtained in the AlexNet classifier at the resting state (Acc: 99.74%), while in the STFT method, a resting state of (Acc: 99.32%) was obtained with the recommended DCNN model.

If we consider the study according to the state of resting, auditory, and visual stimuli, according to Table 1, Table 2 and Table 3, the most successful results were obtained at resting state in all classifier models. While the most successful results after the resting state were obtained in the auditory stimulus situation, the partially less successful ones were in the visual stimulus situation. According to the resting state, the most successful results were obtained in the CWT method with the AlexNet classifier (Acc: 99.74%, Sens: 99.9%, Spec: 99.52%). Regarding the state of auditory stimuli, the most successful results were obtained with the DCNN model recommended in the CWT method (Acc: 99.44%, Sens: 99.04%, Spec: 99.74%). The CWT method and DCNN model showed the most successful performance with respect to the state of visual stimuli (Acc: 98.96%, Sens: 98.24%, Spec: 99.5%).

## 4. Discussion

In this study, a new system based on EEG signal and DL is proposed for the effective and early diagnosis of migraine disease. In the proposed system, images were created in the T-F plane by applying CWT and STFT methods to EEG signals. It has been stated that images can be obtained in the T-F plane from biomedical signals and can be evaluated together with DL models to yield successful results [21]. Studies have also conducted evaluations using CWT and STFT methods [34]. For this reason, CWT and STFT methods were used in this study, and their performances were compared by evaluating them in CNN models. Unlike in ML methods, steps such as feature extraction or feature selection are performed automatically in DL models [11,35]. In this way, faster results can be obtained compared to ML methods. For this reason, three different CNN architectures and the DCNN model that we created were used in the classification stage of this study. As a result of the study, the state, method, and classifier model that showed the most successful performance were determined. For this reason, in addition to the AlexNet, SqueezeNet, ResNet50, and CNN models that are frequently preferred in the studies, the DCNN model, whose layers and parameters we adjusted ourselves, was used in the classification phase of the study. Looking at the results in Table 1, Table 2 and Table 3, it is clear that the proposed DCNN model performs successfully and provides accurate results. The proposed model can be improved with different layers or parameters, but we think that it is suitable for similar studies as it is.

EEG-based DL studies are promising and such studies have become increasingly widespread in recent years. It has been stated in the literature that DL-based CAD systems are widely used for the diagnosis of many diseases [35]. It can be seen in the recently published literature that successful studies on neurological diseases make use of EEG signals [22,27,30,34]. However, studies on MD diagnosis using EEG signals with ML and especially DL models are scarce [14,35,36]. Studies on MD diagnosis using EEG signals and DL models seem to be lacking and new studies are needed [11,37]. We reviewed the recent studies on the diagnosis of MD based on EEG signals and ML-DL and compared their results with the results obtained in this study, as seen in Table 4.

As can be seen in Table 4, studies using ML are more common than DL-based studies. Upon examining studies that diagnose MD based on EEG signals and ML [6,10,15,16,36], we identified that some features are extracted from EEG signals and evaluated in ML methods. Among these studies, Aslan [15] achieved the most successful performance in his study which involved a EMD method and RF classifier (Acc: 92.47). Regarding DL, Göker [14] classified MD and HC groups with 95.99% accuracy. When we look at the studies in Table 4, it is clear that there is only one study on visual stimulus, in which EEG signals at rest were mostly used [6]. As far as we know, no such study has been conducted on auditory stimuli. In this study, EEG signals recorded depending on the resting state, visual stimulus, and auditory stimulus were used. In this way, the most successful method and classifier model were determined while the stimulus effect was also examined. As far as we know, this study is the first of its kind. According to the results in Table 4, it is clear that this study performed more successfully than similar studies in the literature. The positive aspects of this study are as follows:

1. We think that this study is very comprehensive. In this study, besides the EEG signal and DL model-based automatic diagnosis of MD, the effect of three conditions (R-A-V) was also investigated. In addition, a single T-F method was not used. Their performances were compared by applying CWT and STFT methods, both of which are widely preferred in other studies. In addition to the CNN architectures that are frequently used in studies in the literature, the performances of these classifiers were compared by creating our own DCNN model. To our knowledge, we think that this study is the first in the literature do this.
2. Although EEG- and DL-based studies have been conducted on the diagnosis of MD [14,35], this study is the first of its kind. From our research, it became clear that there are few studies on the diagnosis of migraine disease, with DL-based studies being especially lacking. Due to this, we think that this study is important in terms of filling this gap in the literature.
3. We consider it an advantage that the dataset used in this study is new and has not been used much.
4. According to the studies conducted by evaluating the dataset used in this study [10,14,15], the proposed study performed more successfully (Acc: 99.74%, Sens: 99.9%, Spec: 99.52%).
5. It is known that the CWT method gives more detailed features than other T-F methods and is preferred in other studies [35]. Upon examining the results obtained in this study, the CWT method was found to be more successful, which is in alignment with the existing literature.
6. It has been seen that the DCNN model proposed in the study gives close results or is partially more successful with the CNN architectures that are widely preferred in the literature. We think that the proposed model can be evaluated in future studies on different migraine data or on the diagnosis of neurological diseases.
7. As far as we know, this study is the first study regarding EEG signals and DL-based diagnosis of MD based on the resting state and visual and auditory stimuli. According to the results obtained in this study (Table 1, Table 2 and Table 3), we think that the proposed system has potential in the diagnosis of MD.

In addition to the positive aspects of this study, we think that there are also some limitations. These limitations are as follows:

1. Studies on MD diagnosis using EEG signals and DL models are very scarce. For this reason, there have not been many studies in which we can compare the results obtained in this study.
2. EEG-, ML-, and DL-based studies on the diagnosis of MD are scarce, and there is no such study on the stimulus effect as far as we know. For this reason, although the results we obtained in this study are promising, there is no study in which we can compare the stimulus effect.
3. We think that the number of data used in the study was sufficient. However, more data could have improved our results.
4. The method used in the study and the proposed model could not be tested because there was no other migraine data. The performance of the proposed method and model can be compared by using different migraine data in the future.

## 5. Conclusions and Future Work

**Figure 6:**
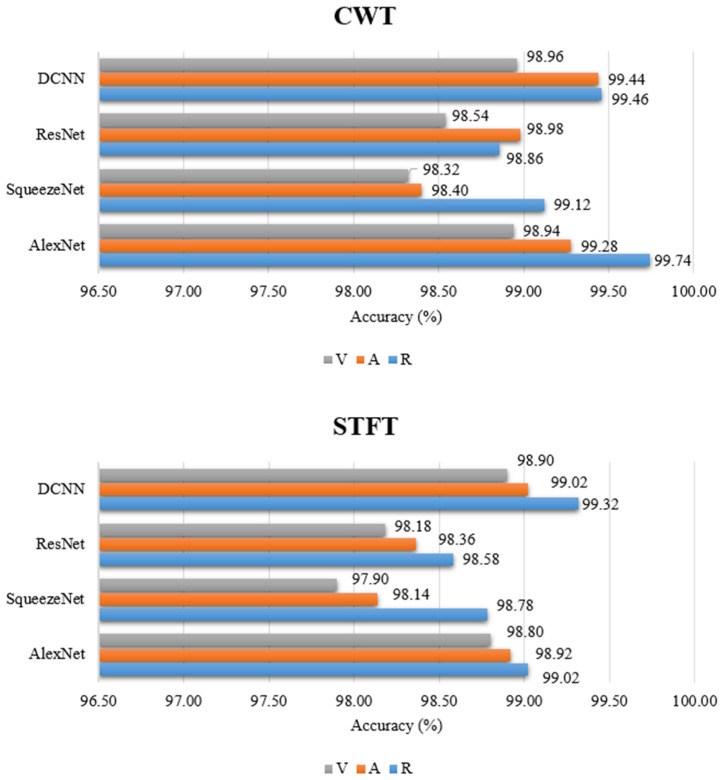
Classification results obtained by applying CWT and STFT methods to deep learning architectures.

Although migraine is one of the most common neurological diseases, studies on migraine are lacking. Especially EEG signal- and DL-based studies on MD diagnosis are very few. For this reason, new studies are needed. One of our biggest motivations for conducting this study was that very few studies of this type exist. Early diagnosis of MD can be difficult and time-consuming for specialists. For this reason, this study aimed to propose an EEG- and DL-based system that can support specialist physicians for the automatic and early diagnosis of MD. For this purpose, EEG signals of MD and HC groups were examined depending on three conditions. In the study, two different T-F methods were applied and their performances were compared. In the classification phase of the study, their performances were compared using three different CNN architectures and the DCNN model we suggested. In this comprehensive study, stimulus state, method, and a classifier model were used to determine the most successful performance. The results obtained in the study show that the methods and classifier models used can help experts in the early diagnosis of MD. Considering the results in Figure 6, it is thought that the preferred method and classifier models in the study are promising for the diagnosis of MD. In addition to the ready-made CNN architectures that are widely preferred in studies, the DCNN model we created in this study was also used in the classification phase. The performance of our proposed model gave better results than the SqueezeNet and ResNet50 architectures in this study. Similar results were obtained according to the AlexNet architecture. Although the proposed model gave successful results in this study, we think that layer and parameter information should be improved and evaluated in different migraine data in the future. In this way, the performance of the model can be interpreted more accurately. However, we think that the proposed methods and models should be evaluated using different migraine data in the future to more accurately determine their effectiveness. The proposed methods and models could also be considered for use in studies regarding the early diagnosis of different neurological diseases based on EEG.

## Figures and Tables

**Table 1:** Resting state classification results (%).

		CWT		STFT		
Classification Model	Acc.	Sens.	Spec.	Acc.	Sens.	Spec.
DCNN	99.46	99.90	99.08	99.32	99.60	99.06
AlexNet	**99.74**	99.90	99.52	99.02	99.18	98.88
SqueezeNet	99.12	99.18	99.08	98.78	98.56	98.96
ResNet50	98.86	98.64	99.06	98.58	97.82	99.26

**Table 2:** Auditory stimulus status results (%).

		CWT		STFT		
Classification Model	Acc.	Sens.	Spec.	Acc.	Sens.	Spec.
DCNN	**99.44**	99.04	99.74	99.02	98.80	99.22
AlexNet	99.28	98.80	99.66	98.92	97.96	99.66
SqueezeNet	98.40	96.64	99.66	98.14	97.20	98.88
ResNet50	98.98	98.30	99.48	98.36	97.24	99.24

**Table 3:** Visual stimulus status results (%).

		CWT		STFT		
Classification Model	Acc.	Sens.	Spec.	Acc.	Sens.	Spec.
DCNN	**98.96**	98.24	99.50	98.90	97.82	99.74
AlexNet	98.94	99.38	98.62	98.80	98.26	99.22
SqueezeNet	98.32	97.72	98.86	97.90	97.22	98.52
ResNet50	98.54	98.30	98.70	98.18	97.80	98.50

**Table 4:** Comparison of results from existing migraine studies in the literature and the results of this study.

Study	Data Type	Dataset	Best Methods	Best Classifier	Accuracy (%)
Akben et al. [6] (2016)	EEG (V)	30 MD-30 HC	AR Burg	SVM	85
Aslan [10] (2021)	EEG (R)	18 MD-21 HC	TQWT	Rotation Forest	89.6
Göker [14] (2022)	EEG (R)	18 MD-21 HC	Welch	BiLSTM	95.99
Aslan [15] (2022)	EEG (R)	18 MD-21 HC	EMD	RF	92.47
Subaşı et al. [16] (2019)	EEG (R)	15 MD-15 HC	DWT	RF	85.95
Jindal et al. [36] (2018)	EEG (R)	13 MD-13 HC	Permutation Entropy Fractal Dimensions	RF	88
**Proposed study**	EEG (R) EEG (A) EEG (V)	18 MD-21 HC	CWT	AlexNet Proposed DCNN Proposed DCNN	99.74 99.44 98.96

## Data Availability

The data that support the findings of this study are openly available at [https://kilthub.cmu.edu/articles/dataset/Ultra_highdensity_EEG_recording_of_interictal_migraine_and_controls_sensory_and_rest/12636731] (accessed on 13 May 2022).
